# Pyrroloquinoline quinone ameliorates PM2.5‐induced pulmonary fibrosis through targeting epithelial–mesenchymal transition

**DOI:** 10.1111/jcmm.18299

**Published:** 2024-04-13

**Authors:** Chia‐Chia Chao, Sheng‐Yen Hsiao, Wan‐Chen Kao, Pei‐Chen Chiou, Chieh‐Chen Huang, Mei‐Ting Wang, Po‐Chun Chen

**Affiliations:** ^1^ Department of Respiratory Therapy Fu Jen Catholic University New Taipei Taiwan; ^2^ Division of Hematology‐Oncology, Department of Internal Medicine Chi Mei Medical Center Tainan Taiwan; ^3^ Institute of Clinical Medicine, College of Medicine National Cheng Kung University Tainan Taiwan; ^4^ Department of Nursing Chung Hwa University of Medical Technology Tainan Taiwan; ^5^ Department of Life Sciences National Chung Hsing University Taichung Taiwan; ^6^ Division of Physical Medicine and Rehabilitation Fu Jen Catholic University Hospital Taipei Taiwan, ROC; ^7^ School of Life Science National Taiwan Normal University Taipei Taiwan; ^8^ Translational medicine center Shin‐Kong Wu Ho‐Su Memorial Hospital Taipei Taiwan; ^9^ Department of Medical Research China Medical University Hospital, China Medical University Taichung Taiwan

**Keywords:** EMT, PM2.5, PQQ, pulmonary fibrosis

## Abstract

Pulmonary fibrosis is a lung disorder affecting the lungs that involves the overexpressed extracellular matrix, scarring and stiffening of tissue. The repair of lung tissue after injury relies heavily on Type II alveolar epithelial cells (AEII), and repeated damage to these cells is a crucial factor in the development of pulmonary fibrosis. Studies have demonstrated that chronic exposure to PM2.5, a form of air pollution, leads to an increase in the incidence and severity of pulmonary fibrosis by stimulation of epithelial–mesenchymal transition (EMT) in lung epithelial cells. Pyrroloquinoline quinone (PQQ) is a bioactive compound found naturally that exhibits potent anti‐inflammatory and anti‐oxidative properties. The mechanism by which PQQ prevents pulmonary fibrosis caused by exposure to PM2.5 through EMT has not been thoroughly discussed until now. In the current study, we discovered that PQQ successfully prevented PM2.5‐induced pulmonary fibrosis by targeting EMT. The results indicated that PQQ was able to inhibit the expression of type I collagen, a well‐known fibrosis marker, in AEII cells subjected to long‐term PM2.5 exposure. We also found the alterations of cellular structure and EMT marker expression in AEII cells with PM2.5 incubation, which were reduced by PQQ treatment. Furthermore, prolonged exposure to PM2.5 considerably reduced cell migratory ability, but PQQ treatment helped in reducing it. In vivo animal experiments indicated that PQQ could reduce EMT markers and enhance pulmonary function. Overall, these results imply that PQQ might be useful in clinical settings to prevent pulmonary fibrosis.

## 1 INTRODUCTION

Pulmonary fibrosis is a chronic lung disease that results in irreversible damage and an average life expectancy of 2–5 years post‐diagnosis. The condition is characterized by the abnormal accumulation of extracellular matrix (ECM), leading to scar tissue formation, increased tissue stiffness and gradual replacement of healthy lung tissue with collagen‐rich fibrotic tissue. This substitution causes a decline in pulmonary elasticity and compliance, resulting in lasting respiratory impairment.^1^ Type II alveolar epithelial cells (AEII) are involved in the progression of pulmonary fibrosis. These cells generate surfactant, which decreases surface tension and maintains lung stability. Additionally, they have vital functions in repairing tissues and modulating immunity within the lungs.^2^, ^3^ The AEII cells hold immense importance in repairing damaged lung tissues, primarily by their ability to undergo proliferation and migration towards the provisional ECM, eventually differentiating into type I alveolar epithelial cells. This distinctive role of AEII cells has earned them recognition as protectors of the alveoli.^4^ According to a recent study, the recurrence of damage to AEII cells is believed to be a significant contributor in the development of pulmonary fibrosis.^5^

In addition to salts and organic components, PM2.5 comprises of toxic heavy metals and chemical compounds that tend to accumulate in the respiratory system upon inhalation. Consequently, this fine particulate matter not only affects the lungs but can also have detrimental effects on other vital organs within the body.^6^ Long‐term exposure to PM2.5 has been associated with heightened susceptibility to various grave respiratory illnesses, such as lung cancer, chronic obstructive pulmonary disease (COPD) and pulmonary fibrosis.^7^ According to a recent study, PM2.5 has been found to increase the activation of TGF‐β in AEII cells leading to its involvement in the development of pulmonary fibrosis.^8^ According to another animal investigation, the inhalation of nanoparticles results in pulmonary inflammation and fibrosis.^9^ A comprehensive study using both in vitro and in vivo data outlines the impact of PM2.5 in pulmonary fibrosis.^10^ The results demonstrate that PM2.5 induces profibrotic epithelial–mesenchymal transition (EMT) in bronchial epithelial cells and triggers the differentiation of pulmonary fibroblasts.

PQQ, a redox agent with vitamin‐like properties, is found in bacterial quinoprotein dehydrogenases and lacks covalent bonding. This substance displays significant antioxidant activity and can be detected ubiquitously in soil and flora, and is present to some degree within animal tissues and human milk.^11^ PQQ has been observed to have the capacity for removing reactive oxygen species (ROS) in bacteria and protecting mitochondria from oxidative damage, particularly when reductants are present. Moreover, it exhibits a significant improvement of its antioxidant efficacy even after just one administration in humans.^12^, ^13^ A previous report has showed that PQQ plays a role in diverse biological processes linked to cognitive capabilities, immune reactivity and protection against ischemic occurrences affecting both the heart and nervous system.^14^ A recent report shows that PQQ reduces renal fibrosis by improving mitochondrial function, reducing ROS production and inhibiting NF‐κB/pyroptosis signalling in diabetic nephropathy and hyperglycemia.^15^

Our current investigation has revealed the effectiveness of PQQ in preventing pulmonary fibrosis that results from prolonged exposure to PM2.5. By inhibiting AEII cell migration when exposed to PM2.5, PQQ demonstrated its efficacy in targeting EMT as indicated by changes observed in cellular morphology and expression levels of EMT markers. Notably, our animal study affirmed that administering PQQ led to a reduction in EMT markers and an improvement in pulmonary function among animals subjected to PM2.5 exposure. We conducted this study with the objective of exploring how regulating EMT using PQQ could mitigate pulmonary fibrosis while enhancing its clinical utility.

## 2 MATERIALS AND METHODS

### 2.1 Materials

The materials used for cell culture—F‐12 and DMEM/F‐12 media, fetal bovine serum (FBS), L‐glutamine solution, 100X Insulin‐Transferrin‐Selenium (ITS‐G) and 100X Penicillin–Streptomycin solution—were obtained from Invitrogen (Carlsbad, CA, USA). Antibodies which target collagen vimentin, snail, 1A and β‐actin were procured from Cell Signaling Technology (Danvers, MA, USA). The plastic dishes and well plates, which were employed in cell culture, were procured from Greiner Bio‐One (Frickenhausen, Germany). The PVDF membrane and the Immobilon Western Chemiluminescent HRP Substrate, which were employed in western blot analysis, were provided by Millipore (Billerica, MA, USA). All inhibitors of chemicals were procured from Sigma‐Aldrich. (St. Louis, MO, USA).

### 2.2 Cell culture

The MLE‐12 alveolar type II epithelial cells were obtained from the American Type Culture Collection (ATCC) (ATCC; Manassas, VA, USA). These cells were supplied using a full DMEM/F‐12 medium comprising 2% FBS, ITS‐G, β‐oestradiol (10 nM), hydrocortisone (10 nM), glutamine (2 mM), HEPES (20 mM) and penicillin–streptomycin. The cell cultures were maintained under controlled conditions at 37°C with an atmospheric CO_2_ concentration of 5%. To ensure optimal growth conditions for these cells, the media was replenished every 3 days while subculturing was carried out when they achieved a confluency rate of over 90%. The MLE‐12 cell cultures were cultivated in 6‐well plates and subsequently exposed to PM2.5 at a concentration of 25 g/mL, imitating prolonged exposure effects. Upon completion of incubation cycles, the cells were identified as MLE‐12 p30. In order to establish an appropriate control group for comparison purposes, original cells underwent subculturing over a period of 30 generations.

### 2.3 Scatter assay

To observe in vitro cellular morphological changes associated with EMT, a cell scatter assay was conducted using MLE‐12 cells. A 24‐well culture plate housing 0.5 × 10^4^ cells per well was cultured for 2 days to establish small clusters of cells. Following this, the cells were subjected to designated PM2.5 concentrations over a duration of 1 week. Alterations in colony morphology were examined by capturing photomicrographs under magnification optics during the experimentation period.

### 2.4 Western blot analysis

To perform western blotting, 6‐well plates were used to seed the cells. RIPA buffer was utilized to extract total protein from treated cells. The concentration of protein was measured through the Pierce BCA Protein Assay Kit (Thermo Fisher Scientific) and analysed using SDS polyacrylamide gel electrophoresis (PAGE). Subsequently, proteins separated via SDS‐PAGE were transferred onto Immobilon PVDF membranes for further analysis. The blotting procedure involved initial blocking using 5% BSA for a duration of 1 h at room temperature. Subsequently, primary antibodies were incubated (1:1000 for others and 1:5000 specifically for β‐actin) on the blot also at room temperature for another hour before incubation with secondary antibodies (at a concentration of 1:1000) following which signals on the blots were detected through Immobilon Western Chemiluminescent HRP Substrate from Millipore and captured via UVP's ChemiDoc‐It Imaging System (UVP, Upland, CA, USA). The signals present on blots were quantified using ImageJ software (National Institutes of Health, USA), and protein expression levels were adjusted to β‐actin as reference.

### 2.5 Long‐term PM2.5 exposure in animal model

The animal care guidelines set by the Institutional Animal Care and Use Committee of Shin‐Kong Wu Ho‐Su Memorial Hospital (Taipei, Taiwan) were followed in conducting the in vivo studies. The study involved C57BL/6J mice purchased from NARLabs (Taipei, Taiwan), which were grouped into three: a control group that received 50 μL of PBS, a treatment group that was administered 50 μL of PM2.5 at 10 mg/kg dosage rate and another treatment group given the same dose while also receiving PQQ at 5 mg/kg through intraperitoneal injection. The dosage of PQQ used in the current study was based on a previous study, which used a dosage of 5 mg/kg in mice.^16^ Additionally, the dosage was also calculated using a practice guide for dose conversion between animals and humans, resulting in approximately 4.1 mg/kg for mice, in accordance with the daily suggestion of 20 mg for human adult.^17^ Following 3 months of intranasal administration, twice a week, the mice were humanely euthanized to harvest their lungs which were fixed using formalin solution (10%). The obtained lung samples underwent further processing utilizing Masson's trichrome stain technique as well as immunohistochemistry staining procedure.

### 2.6 Pulmonary function test

To induce anaesthesia, Zoletil (40 mg/kg) mixed with Rompun (10 mg/kg) was administered via intraperitoneal injection to the mice. Afterwards, tracheotomy was performed and endotracheal intubation was carried out followed by stabilization of the tube in the trachea. The Buxco Pulmonary Function Test (BUXCO; Wilmington, NC, USA) was connected during tracheal intubation procedure to measure dynamic pulmonary compliance (Cdyn).

### 2.7 IHC stain

Following the sacrifice of mice, their lung tissues were collected and preserved in formalin solution (10%). After fixation, dehydration was achieved by employing a mixture of xylene and ethanol. The tissue samples underwent embedding into paraffin blocks, which were then sliced to obtain sections with a thickness measuring 5 μm each. Subsequently, these sections were subjected to incubation using primary antibodies diluted at a ratio of 1:100 for 1 h under room temperature conditions. Signal detection within these sections was achieved via employment of the NovoLink Polymer Detection Systems kit (Leica Biosystems, Wetzlar, Germany) according to instructions provided by the manufacturer. The staining results were photographed across three random fields for each slide analysed.

### 2.8 Statistical analysis

All reported values represent the mean ± standard deviation of multiple independent experiments. Statistical comparison between two samples was carried out using Student's *t*‐test. For statistical comparisons involving more than two groups, one‐way analysis of variance (ANOVA) was performed, followed by the Fisher's least significant difference (LSD) post hoc test. A *p* value of less than 0.05 was considered to be statistically significant in all cases.

## 3 RESULTS

### 3.1 PQQ suppresses the upregulation of collagen expression in AEII cells induced by PM2.5 exposure

**FIGURE 1:**
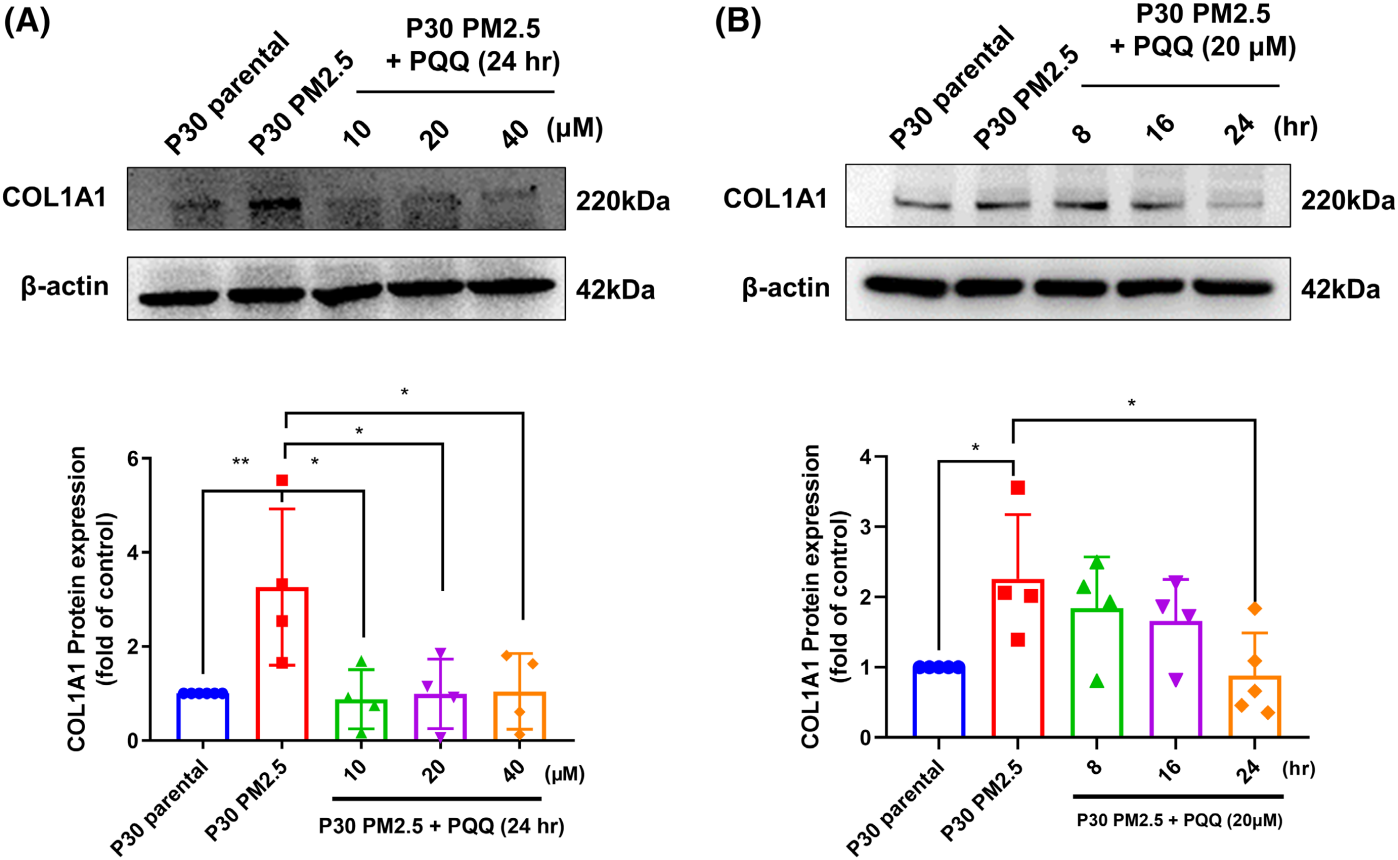
Pyrroloquinoline quinone (PQQ) treatment suppresses the expression of collagen induced by PM2.5 in AEII cells. MLE‐12 AEII cells were exposed to PM2.5 for 30 passages (P30‐PM2.5), and the other was kept for the same duration of 30 passages as a control (P30‐parental). (A) The P30‐PM2.5 cells were then treated with different concentrations of PQQ (10, 20 and 40 μM) for 24 h. (B) The P30‐PM2.5 cells were then treated with PQQ (20 μM) for different time courses (8, 16 and 24 h). Total cell lysates were collected and analysed by western blot to measure the expression of COL1A1, with β‐actin serving as the internal control. The results of the western blot analysis are presented in the lower panel. The results are expressed as mean ± SEM, with *p < 0.05 and **p < 0.01 indicating significant differences compared to each group.

In order to evaluate the anti‐fibrotic effect of PQQ on AEII cells that were exposed to PM2.5 for an extended period, the initial step involved assessing collagen 1A1 (COL1A1) expression as it serves as a widely accepted indicator of fibrotic progression.^18^ According to the data presented, prolonged exposure to PM2.5 has been linked with a substantial elevation in COL1A1 expression. Additionally, administration of PQQ was observed to significantly reduce COL1A1 protein expression dose (Figure 1A) and time (Figure 1B) dependently in AEII cells with long‐term exposure of PM2.5. Treatment with PQQ also inhibited COL1A1 mRNA expression (Figure S1). The result shows that PQQ has the ability to reduce the production of collagen that is induced by PM2.5 exposure.

### 3.2 PQQ effectively inhibits the PM2.5‐induced EMT in AEII cells

According to reports, Type I collagen has been found to facilitate EMT in various situations.^19^, ^20^ Therefore, we conducted an investigation into the potential of PQQ for counteracting PM2.5‐induced EMT in AEII cells.

**FIGURE 2:**
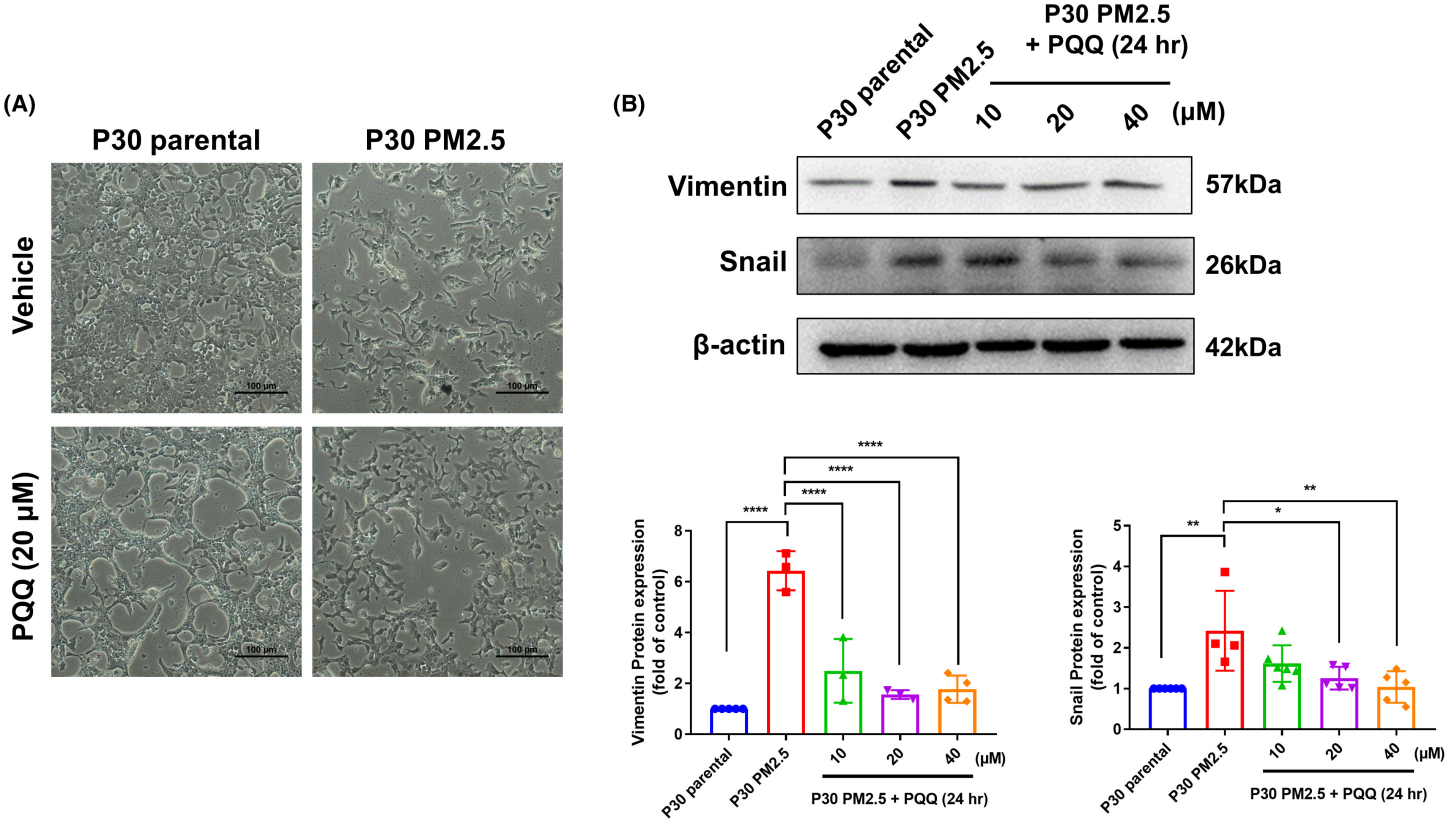
Pyrroloquinoline quinone (PQQ) is effective in reversing the process of EMT induced by PM2.5 in AEII cells. (A) The P30‐PM2.5 cells underwent a 24‐h treatment of PQQ (40 μM), and their cellular structure was observed through a microscope. (B) The P30‐PM2.5 cells received varying amounts of PQQ (10, 20 and 40 μM) for a duration of 24 h. Following this, the expression levels of Vimentin and Snail were measured through western blot analysis using total cell lysates collected from the treated cells. β‐Actin was utilized as an internal control during the process. The outcomes obtained from quantifying data generated by western blot analysis are illustrated in the right panel. The results are expressed as mean ± SEM, with *p < 0.05 and **p < 0.01 indicating significant differences compared to each group.

The findings indicate that exposure to PM2.5 leads to a shift in the morphology of AEII cells towards a mesenchymal‐like appearance, which is reversed by treatment with PQQ as demonstrated in Figure 2A. Furthermore, an examination of EMT markers revealed an elevation in mesenchymal markers like Vimentin and Snail following exposure to PM2.5; however, this impact was reversed after administering PQQ therapy (Figure 2B). According to these findings, PM2.5‐induced EMT in AEII cells can be reversed by PQQ treatment.

### 3.3 PQQ decreases the migratory capacity of AEII cells triggered by prolonged exposure to PM2.5

**FIGURE 3:**
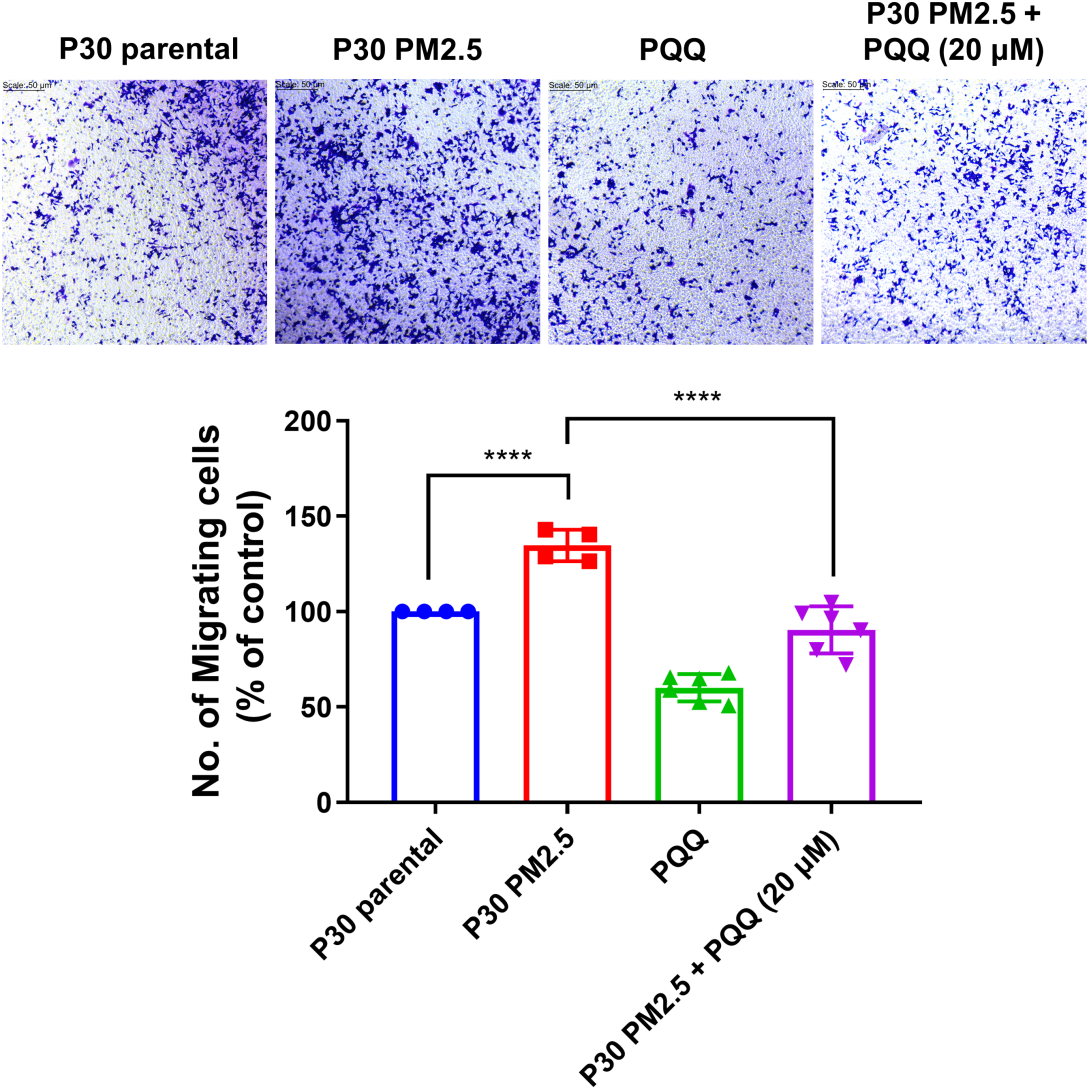
Pyrroloquinoline quinone (PQQ) inhibits the migration capacity of AEII cells stimulated by prolonged exposure to PM2.5. To evaluate the migration capacity of P30‐PM2.5 cells, the cells were treated with 40 μM of PQQ and subjected to the Transwell migration assay for a duration of 24 h. The quantity of migrated cells was determined by capturing images using a microscope and analysing them in the lower panel. The results are expressed as mean ± SEM, with *p < 0.05, **p < 0.01 and ***p < 0.001 indicating significant differences compared to each group.

Subsequently, we assessed the migratory capacity of AEII cells as an indicator of EMT induction.^21^ The results indicated that exposure to PM2.5 led to an induction in the migratory potential of AEII cells. Nevertheless, PQQ treatment was found to effectively attenuate this elevated migratory capability, as illustrated in Figure 3. This indicates that PQQ may prevent AEII cell migration induced by PM2.5 exposure.

### 3.4 PQQ ameliorates pulmonary function caused by prolonged exposure to PM2.5

**FIGURE 4:**
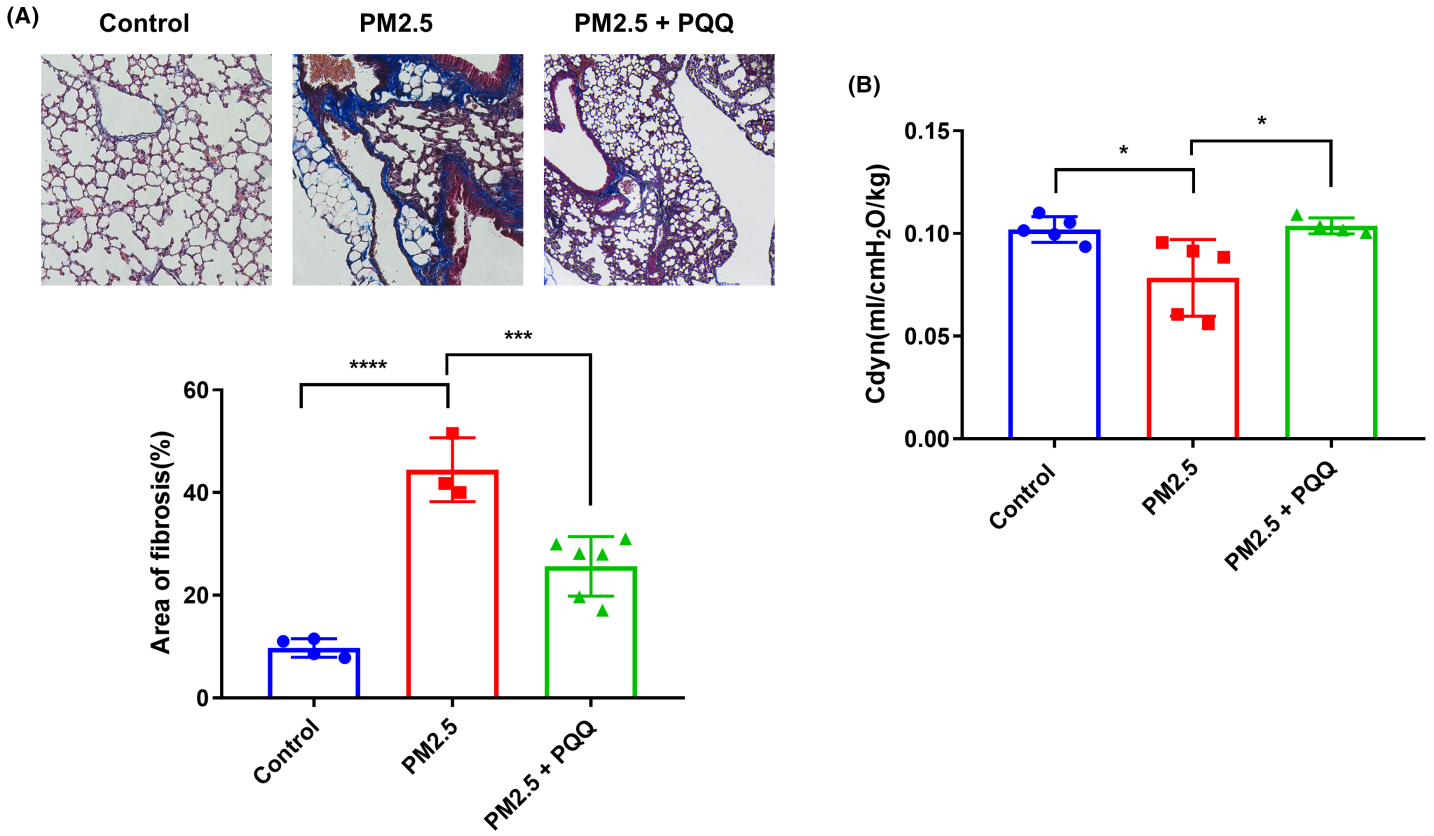
Pyrroloquinoline quinone (PQQ) has the potential to enhance lung function in mice exposed to PM2.5 for an extended period of time. (A) The mice were administered PBS control and PM2.5 (25 μg/mice) twice a week for 12 weeks via their nasal cavities. The treatment group was given PQQ through intraperitoneal injection at a dosage of 5 mg/kg. To assess collagen accumulation, lung specimens were subjected to Masson's trichrome staining and imaging, where blue stain indicates collagen and red highlights cell nuclei. The percentage of the total area with blue staining was used as an indicator of the amount of collagen accumulation. (B) Dynamic pulmonary compliance (Cdyn) in various groups of mice. The results are expressed as mean ± SEM, with *p < 0.05 and ^****^ *p* < 0.0001 indicating significant differences compared to each group.

**FIGURE 5:**
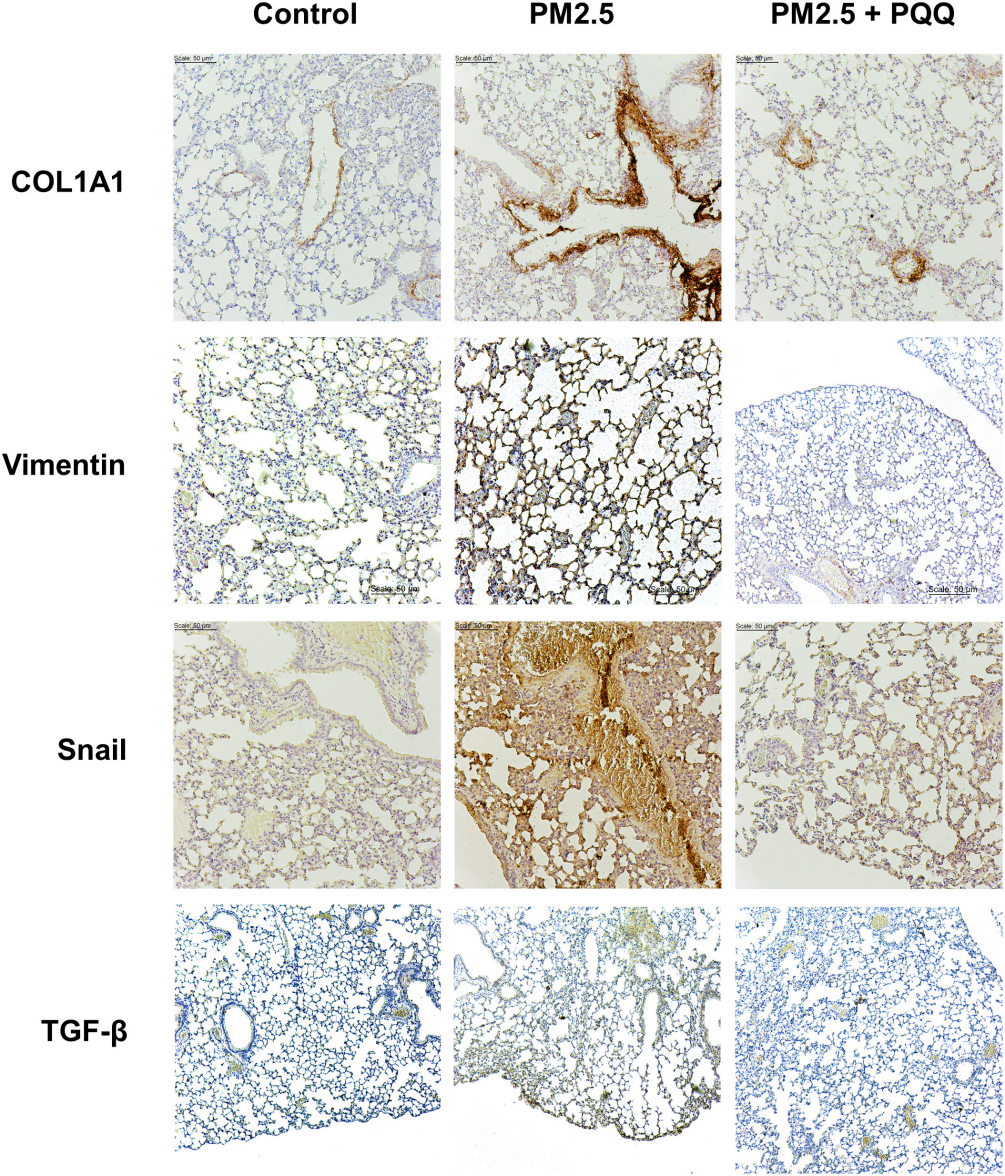
Pyrroloquinoline quinone (PQQ) suppressed the accumulation of collagen and EMT caused by PM2.5 in an animal model. The lung samples from the mice were examined using IHC staining for COL1A1, Vimentin, Snail and TGF‐β. The specimens belonged to three groups: a control group treated with PBS, another group exposed to PM2.5 (10 mg/kg) and a third group given PM2.5 (10 mg/kg) in combination with PQQ (5 mg/kg). The intensity of the stain was captured through microscopic imaging.

To evaluate the potential protective benefits of PQQ against pulmonary fibrosis, an animal experiment was performed. During this study, mice were exposed to PM2.5 through intranasal instillation for a period of 3 months and then treated with either intraperitoneal injections of PQQ or PBS as a control. Notably, the Masson's trichrome stain showed an increase in collagen deposition in the lungs after exposure to PM2.5, but PQQ administration successfully reduced the accumulation of collagen caused by PM2.5 exposure (Figure 4A). According to established knowledge, pulmonary fibrosis is characterized by a decline in lung function. To thoroughly examine the impact of PQQ on PM2.5‐induced lung dysfunction, we conducted a dynamic pulmonary function test. The results showed that the group exposed to PM2.5 had significantly reduced dynamic compliance (Cdyn) compared to the control group (Figure 4B). However, administering PQQ considerably improved lung compliance in mice subjected to PM2.5 exposure, which suggests that it may be beneficial for ameliorating PM2.5‐related lung dysfunction. Following this, an examination was conducted to analyse the induction of collagen and EMT markers in vivo. As shown in Figure 5, it was discovered that COL1A1, Vimentin, Snail and TGF‐β expression was increased in those exposed to PM2.5 compared to the control group. Nevertheless, administering PQQ resulted in a significant reduction in these markers when compared with the PM2.5‐exposed group. These results present persuasive evidence indicating that PQQ provides protection against pulmonary fibrosis caused by PM2.5 exposure and improves lung function in vivo.

## 4 DISCUSSION

There is strong evidence that prolonged and consistent exposure to PM2.5, a type of fine particulate matter, can lead to higher rates of various lung conditions including COPD, pulmonary fibrosis and lung cancer. These ailments usually appear after a considerable period ranging from 5 to 15 years.^7^ A previous study suggested that PQQ, a natural antioxidant, has anti‐fibrotic effects in liver fibrosis by mitigating oxidative stress, suppressing hepatic inflammation and modulating hepatic stellate cell activation.^22^ Furthermore, PQQ has beneficial effects on renal fibrosis in conditions of diabetic nephropathy and hyperglycemia. PQQ was found to alleviate mitochondrial dysfunction, reduce ROS production and inhibit the activation of the NF‐κB/pyroptosis pathway, leading to a potential therapeutic intervention for diabetic nephropathy and hyperglycemia‐induced renal fibrosis.^15^ However, the comprehensive investigation of the mechanism by which PQQ averts PM2.5‐triggered pulmonary fibrosis via EMT remains insufficiently explored. Our research revealed that PQQ possesses the capability to impede the progression of pulmonary fibrosis triggered by chronic PM2.5 exposure. PQQ was observed to inhibit the migration of AEII cells when exposed to PM2.5 by modulating EMT, as evidenced by alterations in cell morphology and EMT marker expression. Furthermore, our animal study results demonstrated that PQQ reduced EMT markers and ameliorated pulmonary function in animals exposed to PM2.5. A previous review has revealed that PQQ can form different derivatives under different pH conditions,^23^ which may occur inside the gastrointestinal tract during uptake. In the current study, we conducted intraperitoneal administration in the animals to evaluate the effects of PQQ engagement, focusing on its pharmacological and proof‐of‐concept aspects rather than on the properties of its pharmacokinetics for clinical translation.^24^ This study aimed to investigate the impact of PQQ on EMT regulation and its potential as a preventive reagent against pulmonary fibrosis, with the ultimate goal of enhancing its clinical utility.

PQQ has been found to effectively impede the process of EMT in human tongue squamous cell carcinoma cells. The inhibition is accompanied by an increase in the generation of ROS and a modification of EMT‐related proteins, such as the upregulation of E‐cadherin and downregulation of Vimentin and Snail. Furthermore, PQQ curbs activity within the NF‐κB signalling pathway. These findings propose that PQQ may have potential as a therapeutic agent for preventing EMT and inhibiting tumour progression in tongue squamous cell carcinoma.^25^ ROS have an important function as secondary messengers that control a range of signalling pathways affecting various cellular functions like cell growth, programmed cell death, self‐degradation process, movement, genetic material damage response mechanism, inflammation and the ability to withstand drugs.^26^, ^27^, ^28^ According to previous studies, ROS is believed to facilitate EMT in cancer cells, particularly in hypoxia environments. For instance, a previous study propose a mechanism involving the direct role of ROS‐mediated EMT in cancer stem cells (CSCs), specifically in response to ionizing radiation.^29^ Karicheva et al. suggest that the production of ROS, triggered by TGF‐β, activates PARP3 in breast cancer cells. This activation is a key factor for inducing EMT as well as promoting cancer stem cell characteristics.^30^ PQQ has been shown to have various beneficial effects, such as functioning as an essential nutrient, antioxidant, and redox modulator in cell culture experiments and animal models. In the current study, we found that PQQ could abrogate EMT in AEII cells. We also found that PQQ was able to inhibit TGF‐β expression in response to PM2.5 exposure in this animal study. Therefore, TGF‐β and ROS maybe the potential key factors that mediated PQQ's anti‐fibrotic effects. However, the detailed mechanisms should be further investigated in future studies.

## 5 CONCLUSION

The current study revealed that PQQ has the ability to treat pulmonary fibrosis caused by exposure to PM2.5. This discovery presents a new and innovative way of using PQQ in treating this condition.

## AUTHOR CONTRIBUTIONS

**Chia‐Chia Chao:** Conceptualization (equal); data curation (equal); formal analysis (equal); methodology (equal). **Sheng‐Yen Hsiao:** Formal analysis (supporting); methodology (supporting). **Wan‐Chen Kao:** Formal analysis (supporting); methodology (supporting). **Pei‐Chen Chiou:** Formal analysis (supporting); methodology (supporting). **Chieh‐Chen Huang:** Conceptualization (equal); data curation (equal). **Mei‐Ting Wang:** Formal analysis (supporting); methodology (supporting). **Po‐Chun Chen:** Conceptualization (equal); data curation (equal); formal analysis (equal); methodology (equal); writing – original draft (lead); writing – review and editing (lead).

## FUNDING INFORMATION

This work was supported by grants from the National Science and Technology Council (NSTC‐111‐2320‐B‐030‐012‐), Fu Jen Catholic University (FJCU‐110‐A0110007), Shin‐Kong Wu Ho‐Su Memorial Hospital and Fu Jen Catholic University (109‐SKH‐FJU‐04, 110‐SKH‐FJU‐04, 111‐SKH‐FJU‐03) and Chi Mei Medical Center (CLFHR11203, CLFHR11209, CLFHR11237, CLFHR11239).

## CONFLICT OF INTEREST STATEMENT

The authors declare no conflicts of interest.

## Supporting information


Figure S1.

## Data Availability

The data sets used and analysed during the current study are available from the corresponding author upon reasonable request.
