# Supplementary material for: Pyrroloquinoline quinone ameliorates PM2.5‐induced pulmonary fibrosis through targeting epithelial–mesenchymal transition
Source: J Cell Mol Med. 2024 Apr 13;28(8):e18299. doi: 10.1111/jcmm.18299 (PMC11015390; doi:10.1111/jcmm.18299)

Figure S1. **PQQ inhibits collagen expression induced by PM2.5 time dependently in AEII cells.**

The MLE-12 AEII cells were exposed to PM2.5 for 30 passages (P30-PM2.5), and the other was kept for the same duration of 30 passages as a control (P30-parental). (A) The P30-PM2.5 cells were then treated with different concentrations of PQQ (10, 20, and 40 μM) for 24 hours. (B) The P30-PM2.5 cells were then treated with PQQ (20 μM) for different time course (8, 16, and 24 hr). The qPCR analysis was conducted to measure the expression of COL1A1. The results are expressed as mean ± SEM, with *p < 0.05, **p < 0.01, and **p < 0.001 indicating significant differences compared to P30 PM2.5 group.


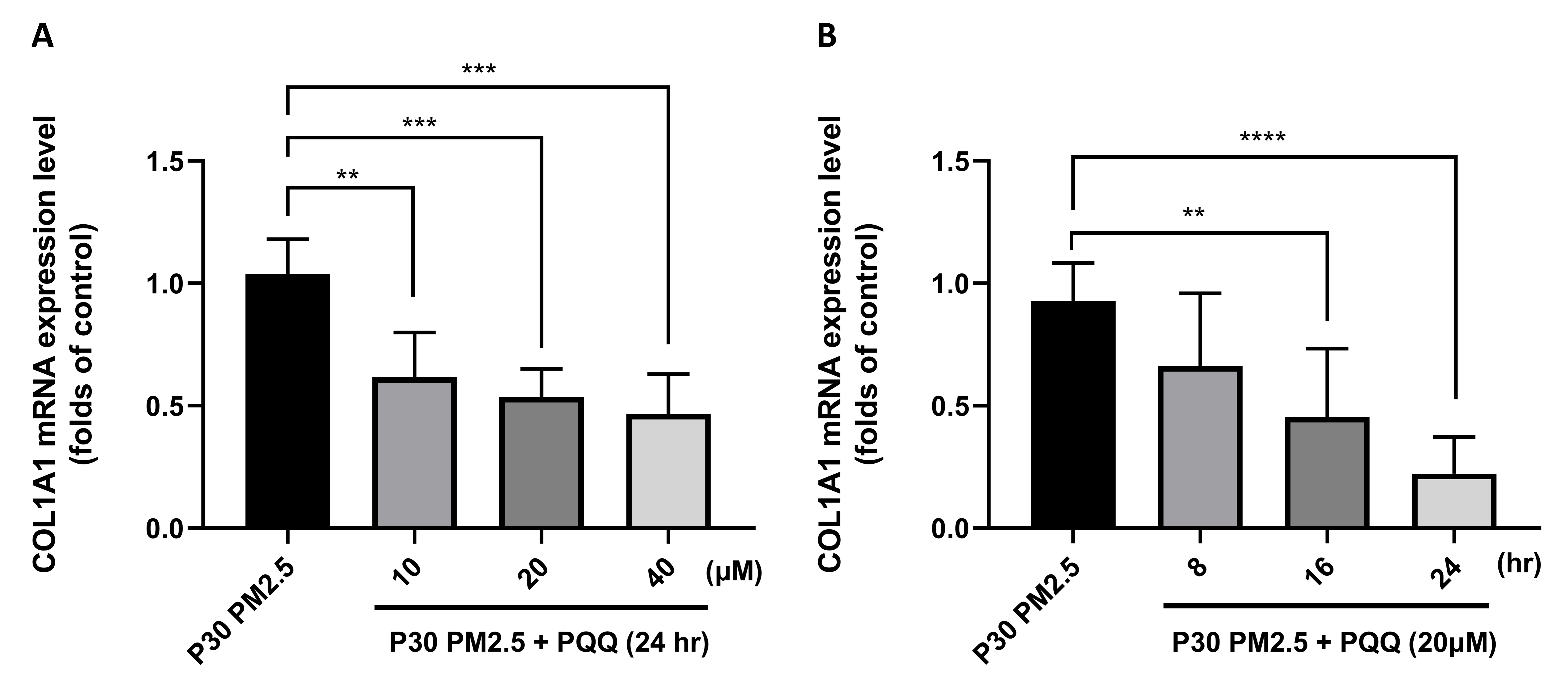

Supplement: Supplementary file 1 — Figure S1. [file JCMM-28-e18299-s001.docx]
